# “Unhealthy = Tasty”: How Does It Affect Consumers’ (Un)Healthy Food Expectations?

**DOI:** 10.3390/foods11193139

**Published:** 2022-10-09

**Authors:** Maija Paakki, Maija Kantola, Terhi Junkkari, Leena Arjanne, Harri Luomala, Anu Hopia

**Affiliations:** 1 Functional Foods Forum, University of Turku, FI-20014 Turku, Finland; 2 School of Marketing and Communication, University of Vaasa, FI-65200 Vaasa, Finland; 3 Technology and Business, Food and Hospitality, Seinäjoki University of Applied Sciences, FI-60101 Seinäjoki, Finland

**Keywords:** unhealthy = tasty (UT) belief, food expectations, tastiness, healthiness, food-related emotions, guilt, health interest, food pleasure orientation

## Abstract

Consumers having a strong unhealthy = tasty (UT) belief are less likely to choose healthy food even though they recognize its health benefits, because they assume healthy food to be unpalatable. The aim of this study was to profile consumers according to their UT belief and specify the strength of the belief among a demographically representative consumer group. The other aim was to investigate the effect of UT belief on expectations of two food products representing either an unhealthy or a healthy image. A total of 1537 consumers participated in the online survey. The scale-based (1–7) mean for UT belief was 3.27 and related positively to male gender and food pleasure orientation and negatively to general health interest. The results indicate that a strong UT belief correlates with positive expectations of unhealthy food and with negative expectations of healthy food. UT belief seemed to increase expected food-associated guilt, but other strong food-related attitudes (health interest with unhealthy food and pleasure orientation with healthy food) reduced this effect. In practice, understanding the relationship between UT belief and personal factors and attitudes, and the importance of this belief to food expectations can assist in finding the tools to encourage consumers towards healthier food choices.

## 1. Introduction

Nowadays, consumers receive a considerable amount of information about healthy diets and are more aware of the benefits of healthy eating. However, despite many public health campaigns aiming to improve dietary habits, recent data suggest that diet quality has remained suboptimal over many years [1,2]. The intake of fruits, vegetables, and whole grains is still lower than recommended, while foods high in energy, fats, free sugars, and salt/sodium are overconsumed [3]. According to the recommendation of the Finnish National Nutrition Council [4], a healthy diet should contain plenty of vegetables, legumes, fruits, and berries, as well as fish, nuts, seeds, and whole-grain cereal products, whereas the intake of meat products and red meat should be restricted. In Finland, the diet has changed remarkably in the direction of the dietary recommendations but still does not entirely reach the food-based dietary guidelines [5]. During the North Karelia project (1972–1997), the intake of salt and total fat was found to have decreased significantly and the consumption of fruit and vegetable to have increased—this intake has now tripled since the 1970s [6,7]. However, in 2017, the recommendation of the National Nutrition Council [4] to eat a minimum of 500 g of vegetables, legumes, fruits, and berries a day was only reached by 14% of men and 22% of women [5]. In addition, the intake of salt and saturated fatty acids is still too high in Finland, especially among men [8].

Although people in general wish to be healthy, they do not always choose healthy food. The key determinants of consumers’ food choices can be categorized as food-internal factors (e.g., sensory features), food-external factors (e.g., physical environment), personal-state factors (e.g., physiological needs, habits), cognitive factors (e.g., knowledge, liking, beliefs), and sociocultural factors (e.g., culture, economic variables) [9]. Goukens and Klesse [10] have categorized food choices as being influenced by three distinct consumer-related forces: lay beliefs (e.g., healthy = non-tasty, healthy = expensive), goals, and habits. Consumers having a strong “unhealthy is tasty” (UT) belief are less likely to consume healthy food because they assume that a healthy diet is relatively unpalatable [2,11]. There is often a contradiction between the desire for a short-term taste and the goal of long-term health, and consumers are found to resolve these conflicts based on their lay beliefs about the healthiness and tastiness of food [11]. Mai and Hoffmann [12] remarked that the objectives of health and taste often conflict, and taste usually prevails in food decision making. Taste is reported to be the most important attribute of food choice [11,12,13,14]. People can form an explicit belief that unhealthy is tasty, but they can also simultaneously hold an implicit intuition associating unhealthiness and tastiness [15,16]. Raghunathan et al. [15] presented the association between unhealthiness and tastiness to operate at an implicit level. However, they found both consumers who reported that they believe that healthiness and tastiness are negatively correlated, and consumers who did not report such a belief, to describe unhealthy items as tastier. The association between unhealthiness and tastiness has been measured at an implicit level (Implicit Association Test), for example [12,15,16], but more recent papers have measured an unhealthy = tasty belief at an explicit level (scale-based measure); for example [11,17,18].

UT belief has been found to be culturally determined [11,16,19]. The US and French cultural contexts have been found to differ substantially with regard to food habits and food attitudes [18]. In the USA, a utilitarian view of food consumption predominates, and US Americans associate food with a biological need and evaluate it from a nutritional and health perspective, whereas in France, food is often associated with pleasure, and “eating well” is related to sensorial and social pleasure [16]. Raghunathan et al. [15] found US Americans to implicitly associate unhealthy food with tastiness, while Werle et al. [16] demonstrated an opposite intuition in France: unhealthy food is associated with bad taste and healthy food with tastiness. Huang and Wu [20] found the food pleasure orientation to diminish the “healthy = less tasty” intuition among US Americans. In addition to cultural contexts, consumers’ educational background and attitudes towards certain aspects of food consumption might affect the “unhealthy = tasty” intuition [18].

Emotions are recognized as an important factor in consumers’ eating behavior and food choice, for example, [21,22,23]. Measuring only the sensory appeal of food products is often discovered to be insufficient [22,24,25]. Therefore, analyzing emotions together with sensory acceptability (liking) of food products has proved to achieve a better understanding of consumers’ food choices [23,25,26,27]. Food is an emotionally charged stimulus, generating both positive (e.g., joy) and negative (e.g., guilt) emotions. In general, food consumption elicits more positive than negative emotions [22,28]. In the context of healthy food consumption, guilt and pleasure are considered important emotions since consumers generally have conflicting food consumption values: the hedonic value of enjoyment and the utilitarian value of staying healthy [29,30]. Feelings of guilt are based on the belief of individuals that they are doing something undesirable or wrong, for example, [29,30]. In general, guilt is aroused during an unpleasant emotional state, and it is considered a negative and complicated emotion impacting consumers’ eating behavior [30,31]. Normally, people seek out feelings that are pleasant and/or rewarding and avoid feelings that are unpleasant and/or unrewarding [21,30]. According to Elder and Mohr [32], especially the conflict between food enjoyment and the achievement of health goals may elicit several distinctly negative emotions, such as guilt. For example, indulging in tasty but unhealthy foods can have a negative effect on health and elicit feelings of guilt. According to Hur and Jang [30], perceived healthiness is found to decrease anticipated guilt, but the effect depends on the consumer’s dietary concerns. Consumers that are highly restrained about their diet are more susceptible to experiencing negative emotions as a result of their heightened state of arousal in the presence of indulgent food [32].

The relationship between UT belief and consumer-dependent factors (demographic factors and food-related attitudes) as well as the effect of UT belief on food expectations, including sensorial, nutritional and emotional expectations, has not yet been adequately studied among demographically (gender, age, education) representative consumer group. Moreover, the prevalence of UT belief among Finnish adults has not been measured, either. In addition, because UT belief is strictly linked to the healthiness of a food product, the effect of the health image of a food product should also be taken into account in research.

**Figure 1:**
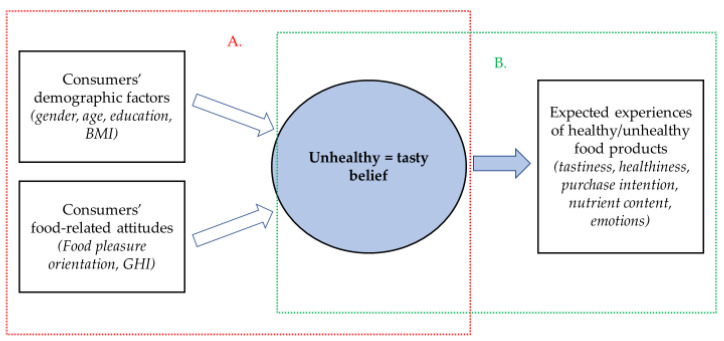
Study scheme. A = The first aim of the study: to specify the strength of unhealthy = tasty belief and to profile consumers according to their unhealthy = tasty belief. B = The second aim of the study: to investigate the effect of unhealthy = tasty belief on the expected experiences of food products that have typically different health images.

The first aim of this study was to specify the strength of UT belief in a representative group of Northern European consumers (Finnish adults) taking into consideration their gender, age, and education. Further, the aim was to profile (demographic factors, food-related attitudes) these consumers according to their UT belief. The second aim was to investigate the effect of UT belief on the expected food experiences (tastiness, healthiness, purchase intention, nutrient content, and emotions) of products having typically different health images. The study scheme is presented in Figure 1.

## 2. Materials and Methods

### 2.1. Study Design 

This study was part of a project exploring the drivers and barriers that influence healthy food choices (REMU—Reformulation of healthier food products in South Ostrobothnia). The online survey (*n* = 1537) was conducted to investigate consumer-dependent factors influencing expected product-related food experiences. The experimental design of the online survey contained six different subgroups (R1–R6) to study the effect of front of pack (FoP) labeling and two different types of interventions (Section S1). The target was to reach ca. 1500 Finnish consumers for the online survey and at least 250 participants in each research group.

In this study, the focus was on UT belief and its effects on food expectations. The first aim was to specify the strength of UT belief among Finnish consumers and to search for individual factors explaining UT belief (Study A). The analyses were conducted among all the participants of the online survey (*n* = 1537). The second purpose of this study was to investigate the effect of UT belief on food expectations. To avoid any interference from the effects of labeling or the intervention, only the subgroup R1 (no FoP, no intervention) was involved in the analyses that investigated the effect of UT belief (Study B; *n* = 262) (Section S1).

The online survey was conducted from 27 January to 9 February 2021. Data were collected using Compusense Cloud software (Compusense Inc., Guelph, ON, Canada). Finnish consumers were recruited via Cint (service platform for consumer panels used in market research, www.cint.com, accessed on 27 January 2021). Data collection and technical implementation were arranged by the consumer research company Aistila Ltd., Turku, Finland.

Participation in the online survey was entirely voluntary, and all participants agreed to an informed consent statement at the beginning of the questionnaire. The data protection statement of the research project was available to all the participants. The entire procedure complied with the guidelines of the Finnish National Board on Research Integrity TENK. According to the statement of the University of Vaasa Human Science Ethics Committee, an ethical review was not required for this online survey.

### 2.2. Participants

A total of 1537 consumers participated in the online survey. They were recruited to form a representative sample of Finnish adults (over 18 years) as regards their gender, age, and education. The demographic factors of the participants in our study (Study A; *n* = 1537) were: gender (50.2% female/49.3% male/0.5% other), age (18–71 years, mean 45 years), and education (54.9% no academic degree/45.1% academic degree). Participants were considered to represent an average group of Finnish consumers: in 2020, the gender divide among Finns was 50.6% female/49.4% male, the mean age was 43.4 years, and 44% of adult Finns (20–69 years) had an academic degree [33].

The corresponding demographic factors of the subgroup investigated in Study B (*n* = 262) were similar to those of Study A: gender (51.1% female/48.5% male/0.4% other), age (19–71 years, mean 45 years), and education (54.6% no academic degree/45.4% academic degree).

### 2.3. Samples

**Figure 2:**
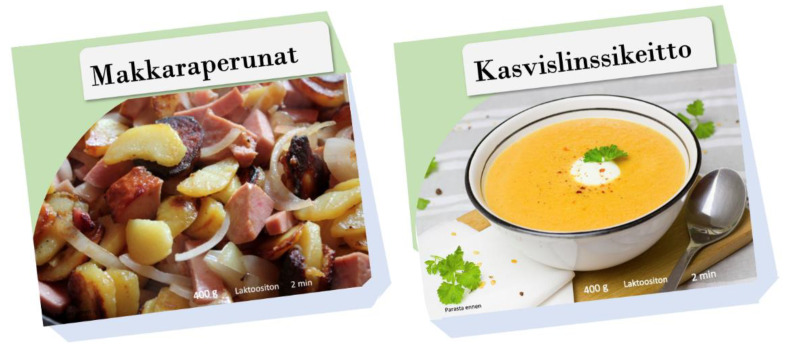
The sample with the unhealthy image (fried potatoes and sausages (Makkaraperunat)) and the sample with the healthy image (vegetable lentil soup (Kasvislinssikeitto)) as evaluated in the online survey.

In the online survey, the participants were asked to form their expectations of two different types of foods based solely on pictures of the food packages. The evaluated food products were typical Finnish ready-to-eat food products. In addition, the packages were designed to resemble the normal packaging of microwave-heated foods sold in Finland (Figure 2).

The food products were selected by the researchers to represent products with different images of health and nutrition content: the fried potatoes and sausages represented a prototypically unhealthy, fatty and salty product, and the vegetable lentil soup prototypically a healthy (a lot of vegetables), less fatty, and less salty product. The assumption that the samples presented different images as regards their health and nutrition content was verified by the results of the expected attributes of the samples (see Figure 4, Section 3.3).

### 2.4. Questionnaire

The questionnaire consisted of three parts: (1). product expectations, (2). consumer profile, and (3). demographic factors (Section S2). In the first part, participants evaluated the expected attributes (a 7-point Likert scale for every attribute anchored with: 1 = strongly disagree, and 7 = strongly agree) for both food products presented in a random order. The expected attributes were: tastiness (2 claims; Cronbach’s α = 0.95 for unhealthy product and 0.95 for healthy product), healthiness (3 claims; Cronbach’s α = 0.91 for unhealthy product and 0.85 for healthy product), purchase intention (3 claims; Cronbach’s α = 0.92 for unhealthy product and 0.91 for healthy product), energy, fat, salt and protein content, and food-related emotions (7 emotions).

The food-related emotions were measured using items: I expect, that tasting and eating this product “… calms and reassures me”, “… bores me, does not interest me”, “… satisfies me”, “… makes me associate it to happy memories of childhood”, “… makes me feel guilt”, “… disgusts me”, and “… makes me feel active and full of energy”. The rationale for using these food-related emotions was to select both positive and negative emotions commonly used in food product experiences [23,25,28]; therefore, out of all the possible (food-related) emotions, those emotions that were assumed to be different when evaluating the samples of this study were selected to be evaluated. The number of emotions was restricted to seven owing to the length of the questionnaire.

The second part of the questionnaire included randomly presented statements (1 = strongly disagree to 7 = strongly agree) defining the food-related attitudes and beliefs of the participants. The statements concerning UT belief [12,15], general health interest (GHI) [34], and food pleasure orientation [20,35] were included (17 statements). The strength of UT belief was assessed by three items (Cronbach’s α = 0.78): “Things that are good for me rarely taste good”, “There is no way to make food healthier without sacrificing taste”, and “Healthy food is usually less tasty” [12,15]. Eight items assessed the participants’ general health interest (GHI) (Cronbach’s α = 0.86) [34], and six items their food pleasure orientation (Cronbach’s α = 0.66) [20,35]. All the Cronbach’s α values were over 0.7 except for the food pleasure orientation. Leaving out the negatively worded item for food pleasure orientation, the Cronbach’s α was improved to 0.70, and this 5-item food pleasure orientation was used in the following data analyses.

The demographic questions (gender, age, and education) were asked in the third part of the questionnaire. In addition, the participants were also asked to report their weight and height; these measurements were used to calculate the participants’ body mass index (BMI): BMI = kg/m^2^ where kg is a person’s weight in kilograms and m^2^ is their height in meters squared.

### 2.5. Data Analysis

Statistical analyses were performed using IBM SPSS Statistics, version 27.0 (IBM Corp. (Armonk, NY, USA), 2020).

Linear regression analysis was used to examine the relationship between UT belief and the demographic factors (gender, age, education, BMI) and attitudes (GHI and food pleasure orientation) (Study A).

To examine which factors (UT belief, gender, age, education, BMI, GHI, food pleasure orientation) best predicted the dependent variables (expected tastiness, healthiness, purchase intention, emotions, nutrient contents), a linear regression analysis was conducted for each dependent variable (Study B). In the regression analysis, the following dichotomous dummy variables were used: gender (0 = female; 1 = male) and education (0 = no academic degree; 1 = academic degree).

The interactions between UT belief and demographic factors (gender, age, education, BMI) and UT belief and attitudes (GHI, food pleasure orientation) were investigated by regression analysis using centered variables together with interaction terms. 

A paired-samples *t*-test was used to compare the mean scores of the different products (products 1 and 2) within participants. An independent-samples *t*-test was used to compare the mean scores of two different groups of participants (participants with either higher or lower UT belief).

## 3. Results

### 3.1. The Strength of UT Belief (Study A)

The scale-based (1–7) mean of UT belief among participants (*n* = 1537) was found to be 3.27 (Md = 3.33; SD = 1.49). Participants represented a Northern European consumer population (Finnish adults) as regards their gender, age, and education.

### 3.2. The Factors Related to UT Belief (Study A)

**Figure 3:**
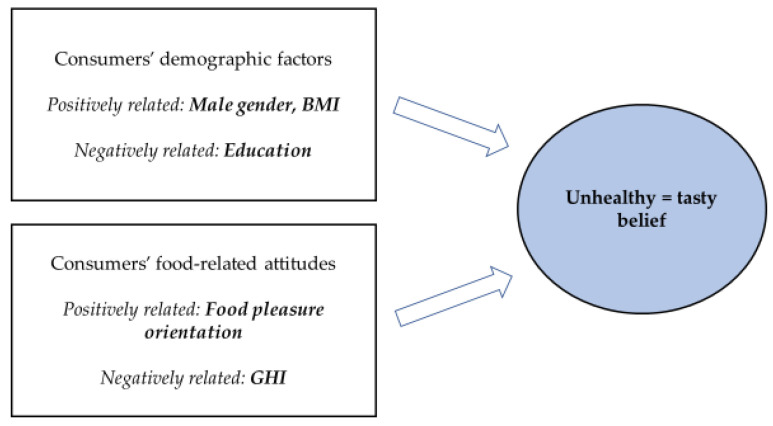
Consumer-related factors (demographic factors and attitudes) related to unhealthy = tasty belief.

The factors found to be related to UT belief are presented in Figure 3.

The regression analysis of UT belief using demographic factors (gender, age, education, and BMI) and attitudes (GHI, Food pleasure orientation) as predictors (Table 1) revealed that GHI (Stand. β = −0.361, *p* < 0.001) and gender (Stand. β = 0.168, *p* < 0.001) were relatively the most important predictors of UT belief: consumers having a stronger UT belief had lower GHI, and a higher number were men. Food pleasure orientation (Stand. β = 0.076, *p* = 0.001) and BMI (Stand. β = 0.048, *p* = 0.042) were positively related to UT belief, while education (Stand. β = −0.057, *p* = 0.015) was found to relate negatively to UT belief.

### 3.3. The Difference between Sample Products Representing Different Health Images (Study B)

**Figure 4:**
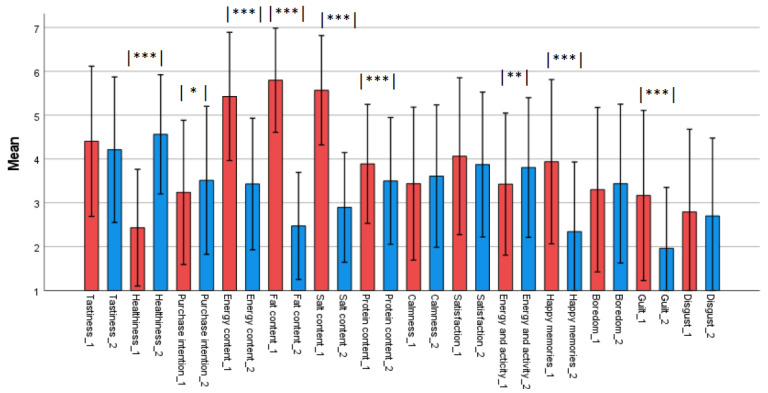
The ratings (*n* = 262) of the expected attributes of the products. Product 1 = fried potatoes and sausages (red bars); product 2 = vegetable lentil soup (blue bars). Significant differences (paired-samples *t*-test) between products 1 and 2 are marked with *** *p* < 0.001, ** *p* < 0.01, and * *p* < 0.05.

The mean of the expected attributes of product 1 representing an unhealthy product (fried potatoes with sausages) and product 2 representing a healthy product (vegetable lentil soup) are presented in Figure 4.

The sample products were selected to represent typically different images of health and nutrient content: the unhealthy product with fat and salt, and the healthy product with less fat and salt and rich in vegetables. The results confirmed the image difference between the sample products. The expected attribute profile of product 1 (unhealthy product; fried potatoes and sausages) was found to be less healthy (*p* < 0.001), and the product had higher ratings for energy, and fat and salt content (*p* < 0.001) compared to product 2 (healthy product; vegetable lentil soup). For the other expected attributes, the two products were rated quite similarly: there were no significant differences between the expected tastiness of the products or the expected emotions: calmness, satisfaction, boredom, and disgust. The only differences between the expected emotions associated with the products were guilt and happy memories of childhood, both of which were rated higher with the unhealthy product.

### 3.4. The Effect of UT Belief on the Expected Attributes of Products Having Either an Unhealthy or a Healthy Image (Study B)

**Figure 5:**
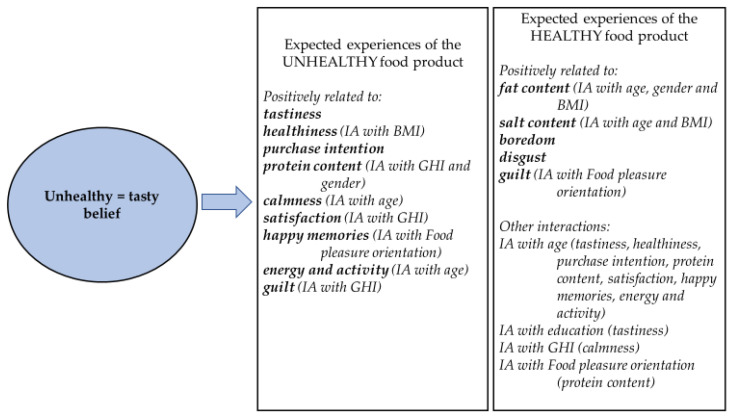
The effect of unhealthy = tasty belief on the expected experiences of the food products having different health image (see Sections S3 and S4). IA = interaction between UT belief and another factor.

The effect of UT belief depended on the health image of the product (Figure 5). With the unhealthy product (fried potatoes and sausages), positive attributes, such as expected tastiness, healthiness, and positive emotions, were positively related to UT belief. With the healthy product (vegetable lentil soup), there was a positive relationship with the expected negative emotions and with the expectations of undesirable nutrients (fat and salt).

The results of the regression analyses revealing predictors of the expected attributes and significant (*p* < 0.050) interactions between UT belief and other factors (gender, age, BMI, education, GHI, Food pleasure orientation) are presented in Section S3 (the unhealthy product) and Section S4 (the healthy product).

The following four sections present the results concerning the effect of UT belief on expected tastiness, healthiness, and purchase intention (Section 3.4.1), on expected nutrient content (Section 3.4.2), on expected food-associated emotions in general (Section 3.4.3), and on expected food-associated guilt (Section 3.4.4).

#### 3.4.1. The Effect of UT Belief on the Expected Tastiness, Healthiness, and Purchase Intention

The linear regression analyses of the expected attributes of the unhealthy product revealed that UT belief was positively related to expected tastiness (Stand. β = 0.167, *p* = 0.007), healthiness (Stand. β = 0.171, *p* = 0.008), and purchase intention (Stand. β = 0.188, *p* = 0.002) (see Table S1). However, with the healthy product, no relationship between UT belief and expected tastiness, healthiness, or purchase intention was found (see Table S5). There were slight UT belief * age interactions with the healthy product as regards expected tastiness (β = −0.021, *p* = 0.017), healthiness (β = −0.011, *p* = 0.010), and purchase intention (β = −0.013, *p* = 0.010). The effect of UT belief was more negative among older participants (older than 44 years).

**Figure 6:**
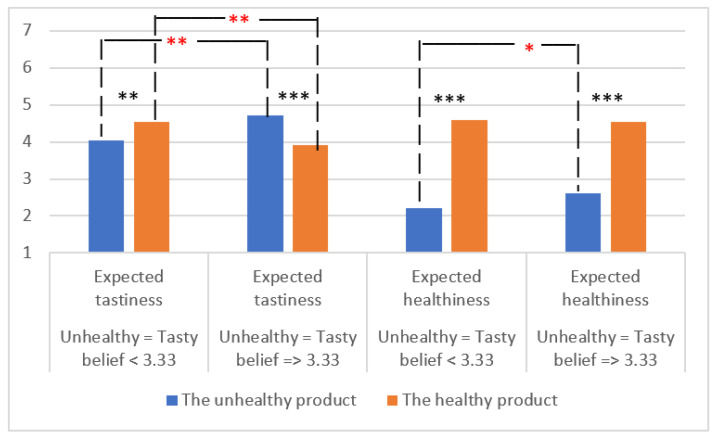
The effect of unhealthy = tasty belief on the expected tastiness and healthiness of the unhealthy (fried potatoes and sausages) and the healthy product (vegetable lentil soup). The differences between expected tastiness and healthiness of different products are marked with *** (*p* < 0.001), ** (*p* < 0.010 (paired-samples *t*-test). The differences between participants with either higher unhealthy = tasty belief (≥3.33) or lower unhealthy = tasty belief (<3.33) are marked with ** (*p* < 0.010), * (*p* < 0.050) (independent-samples *t*-test).

Expected tastiness and healthiness were found to depend both on the health image of the product and the participant’s UT belief (Figure 6). The participants with higher UT belief expected the healthy product to be healthier but less tasty than the unhealthy product, while the participants with lower UT belief expected the healthy product to be both tastier and healthier than the unhealthy product. Moreover, there was a positive relationship between UT belief and expected tastiness and healthiness of the unhealthy product, whereas the relationship was negative with the expected tastiness of the healthy product.

#### 3.4.2. The Effect of UT Belief on Expected Nutrient Content

The linear regression analyses revealed that UT belief was positively related to the expected protein content of the unhealthy product (Stand. β = 0.179, *p* = 0.006) (see Table S2). In addition, significant interaction between UT belief and gender was noticed (see Table S4). Among men, UT belief correlated positively with the expectation of the protein content of the unhealthy product, while no correlation was detected among women.

With the healthy product, UT belief was found to be positively related to the expected fat (Stand. β = 0.248, *p* < 0.001) and salt content (Stand. β = 0.149, *p* = 0.030) (see Table S6). In addition, there was significant UT belief * age interaction with the expected fat (β = −0.011, *p* = 0.003), salt (β = −0.009, *p* = 0.014), and protein content (β = −0.014, *p* = 0.001). The positive effect of UT belief on the expected fat, salt, and protein content was more significant among younger participants (younger than 45 years). Significant interaction was also noticed between UT belief and food pleasure orientation with the expected protein content (β = −0.158, *p* = 0.007) and between UT belief and gender with the expected fat content (β = 0.271, *p* = 0.008) (see Table S8). The positive effect of UT belief on the expected protein and fat content was more significant among participants with a lower food pleasure orientation and among men.

#### 3.4.3. The Effect of UT Belief on Expected Food-Associated Emotions

The linear regression analyses of the unhealthy product revealed that UT belief was positively related to the following expected positive emotions: energy and activity (Stand. Β = 0.228, *p* < 0.001), happy memories of childhood (Stand. Β = 0.199, *p* = 0.002), calmness (Stand. Β = 0.171, *p* = 0.008), and satisfaction (Stand. Β = 0.144, *p* = 0.022) (see Table S3). The results of the analyses of the healthy product were quite different compared to those of the unhealthy product. UT belief was found to be positively related only to the negative emotions associated with the healthy product: disgust (Stand. β = 0.212, *p* = 0.001) and boredom (Stand. β = 0.195, *p* < 0.003) (see Table S7). No relationship was found between UT belief and the positive emotions associated with the healthy product.

With the healthy product, significant interactions between UT belief and age were found when evaluating satisfaction (β = −0.014, *p* = 0.004), happy memories of childhood (β = −0.014, *p* = 0.005), and energy and activity (β = −0.011, *p* = 0.027). Among older (45 years and older) participants, the negative effect of UT belief on expected emotions was more significant compared to those of younger participants.

#### 3.4.4. The Effect of UT Belief on Expected Food-Associated Guilt

UT belief correlated with expected guilt for both the unhealthy (Stand. β = 0.231, *p* < 0.001) and the healthy (Stand. β = 0.303, *p* < 0.001) products (see Tables S3 and S7). With the unhealthy product, expected guilt was the only negative emotion related positively to UT belief, while with the healthy product, other negative emotions such as boredom and disgust were also positively related to UT belief. The mean score of the expected guilt of the unhealthy product was higher: 3.17 (SD = 1.94), compared to 1.96 (SD = 1.39) of the healthy product.

**Figure 7:**
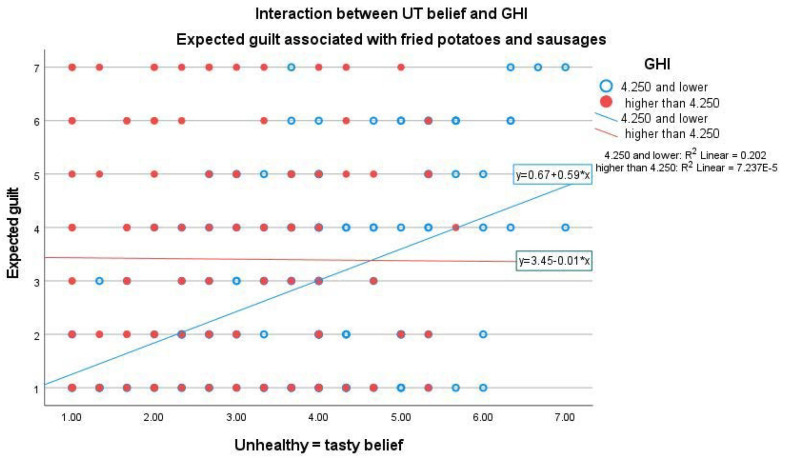
Interaction between UT belief and GHI when rating expected guilt associated with the unhealthy product (fried potatoes and sausages). Linear regression among participants with lower GHI: β = 0.586, *p* < 0.001, CI (95.0%): 0.385–0.787. Linear regression among participants with higher GHI: β = −0.013, *p* = 0.924, CI (95.0%): −0.291–0.264.

With the unhealthy product, male gender and age related negatively to expected guilt, while GHI was found to have a similar positive relationship with guilt compared to UT belief (see Table S1). In addition, significant interaction between UT belief and GHI (β = −0.216, *p* = 0.003) was found when evaluating the expected guilt of the unhealthy product (see Table S4). The positive effect of UT belief on expected guilt was noticed only among participants having lower GHI (Figure 7).

**Figure 8:**
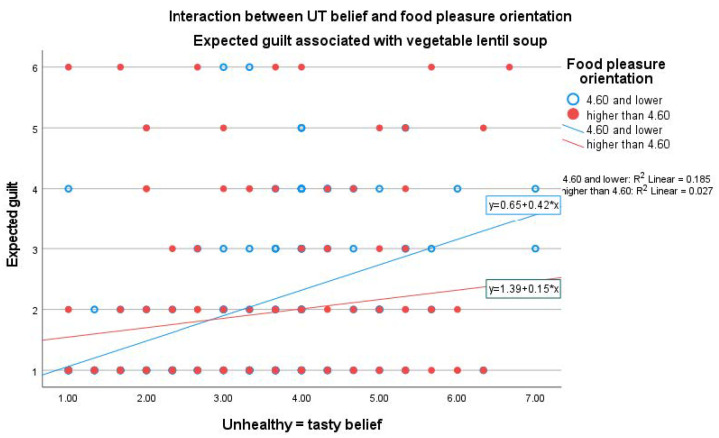
Interaction between UT belief and food pleasure orientation when rating expected guilt associated with the healthy product (vegetable lentil soup). Linear regression among lower food-pleasure-oriented participants: β = 0.418, *p* < 0.001, CI (95.0%): 0.258–0.578. Linear regression among higher food-pleasure-oriented participants: β = 0.154, *p* = 0.050, CI (95.0%): 0.000–0.309.

With the healthy product, UT belief as well as male gender related positively to expected guilt (see Table S5). Significant interaction between UT belief and food pleasure orientation (β = −0.155, *p* = 0.004) was found when evaluating expected guilt (see Table S8). The positive relationship between guilt and UT belief was more significant among participants having a lower food pleasure orientation (Figure 8).

## 4. Discussion

### 4.1. The Strength of UT Belief

The results revealed that the strength of the UT belief we found among Finnish consumers (mean = 3.27) was at the same level as in those studies carried out with heterogeneous samples of consumers in other European countries. In the cross-national survey, Briers et al. [11] measured explicit UT belief using the same three items used in our survey, but with a five-point Likert scale. Consequently, the following results are converted to correspond with the seven-point Likert scale we used in our study. Briers et al. [11] found the mean UT belief values to be 3.22 in Germany (*n* = 730), and 3.78 in the United Kingdom (*n* = 701). Mai and Hoffmann [12] found the average explicit UT belief in Germany (*n* = 560) to be 3.64 (converted from five-point scale to seven-point scale). In Australia, the UT belief measurement was found to be at the same level as in Europe: 3.50 (*n* = 711), but in India and Hong Kong, UT belief was measured as being stronger [11] at 4.72 (*n* = 698) and 4.59 (*n* = 218), respectively. The participants in India and Hong Kong were younger (mean age 32–35 years) compared to the other countries in the survey (mean age 44–54 years). In our study (mean age 45 years), age was not a significant predictor of UT belief, but there was a slight negative correlation between age and UT belief. Cooremans et al. [17] measured explicit UT belief using a similar seven-point Likert scale to our study, but with only two items (according to Raghunathan et al. [15]). Their values for UT belief were slightly lower or similar to our results: 3.11 in the United Kingdom (*n* = 530) and Belgium (*n* = 476), 3.18 in France (*n* = 511), and 3.30 in the United States (*n* = 650). Similar values were also found in studies with fewer participants. The results of a study using French undergraduate students (*n* = 94; mean age 19.6 years) as participants revealed the mean explicit UT belief to be 3.22 (results converted from five-point to seven-point scale) [16]. Heijden et al. [36] measured the strength of UT belief among Dutch citizens with a low socioeconomic position (*n* = 37) and found the median of UT belief to be 2.8 (converted from five-point scale to seven-point scale).

Generally, the strength of explicit UT belief was found to be quite weak (below average: 4 = not agree or disagree with the belief), and the Finnish values were at the same level as in other studies in Europe and the United States. In our study, we had a large and representative group of participants compared to many other studies. The participants (*n* = 1537) in our study represented a typical cross-section of Finnish adult consumers with regard to gender (50.2% female), age (mean 45 years), and education (45% academic degree).

### 4.2. The Factors Related to UT Belief

We found GHI and gender to be the most important predictors of an explicit UT belief, with GHI correlating negatively and male gender positively with the belief. In addition, food pleasure orientation and BMI related positively and education negatively to UT belief. The gender dependence of UT belief was in line with earlier studies. According to Landry et al. [37], beliefs about nutrition and health as well as about food choices and preferences are found to depend on gender. Wardle et al. [38] found gender differences to be attributed to the stronger beliefs of women in healthy eating and their greater involvement in weight control. The study concluded that one reason for men to make less healthy food choices was that health was a less important motivation for men in the food domain when compared to women.

The positive relationship between UT belief and BMI found in our study was also detected by Briers et al. [11], and Mai and Hoffmann [12]. In addition, Cooremans et al. [17] found UT belief to increase the likelihood of an individual becoming obese. Mai and Hoffmann [12] discovered health consciousness to diminish UT belief, which in turn reduced body mass. A similar kind of negative relationship between GHI and UT belief was also detected in our study.

Huang and Wu [20] assumed that people with a high food pleasure orientation would evaluate food mainly from a hedonic perspective, instead of a healthy and utilitarian perspective. Subsequently, their study found the food pleasure orientation to diminish people’s implicit “unhealthy = tasty” intuition. In contrast, we found a positive relationship between the food pleasure orientation and UT belief. The explanation for this difference might be that we specifically investigated the explicit belief, while Huang and Wu [20] explored the implicit “unhealthy = tasty” intuition.

### 4.3. The Effect of UT Belief on the Expectations of Food Products with Different Health Images

The results revealed that the effect of UT belief on expected attributes was dependent on the health image of the food product. Participants with a high UT belief expected the unhealthy product to have more positive attributes (tastiness, purchase intention, protein (desirable nutrient), and more positive emotions (satisfaction, calmness, energy and activity, happy memories of childhood, and even healthiness), and the healthy product to have more negative attributes (undesirable nutrients: fat and salt, negative emotions: disgust and boredom). This positive effect towards unhealthy products probably reflects a halo effect, which is a cognitive bias claiming that a positive impression of a product in one area positively influences feelings in another area, for example, [39,40].

In our study, UT belief was positively related to both expected tastiness and expected healthiness of the unhealthy product, but the same kind of relationship was not detected with the healthy product. By contrast, Haasova and Florack [18] found healthiness and tastiness to relate positively to both unhealthy (snacks) and healthy (drinks) products, but the association was lower with the unhealthy food category. They also noticed that UT belief decreases this positive correlation between healthiness and tastiness. Inconsistency in the results might be explained by the fact that the sample materials in our study (ready-to-eat food products representing typical Finnish lunch foods) were quite different compared to the sample products (snacks and drinks) of Haasova and Florack [18].

### 4.4. The Effect of UT Belief on Expected Food-Associated Guilt

In our study, expected guilt was the only expected emotion related positively to UT belief with both the unhealthy and the healthy product. Consumers having a strong UT belief seemed to feel guilty about food in general, although the reasons for the guilt may differ. Guilt associated with unhealthy food might stem from liking/consuming unhealthy food despite these foods not being good for one’s health, whereas guilt associated with healthy food may come from not liking/consuming healthy food although it is good for your health. For example, Yu et al. [31] noticed that not enjoying eating fresh vegetables makes some consumers feel guilty because they believe they should eat them.

In our study, the mean score of expected guilt associated with the unhealthy product was 3.17 compared to 1.96 with the healthy product, which confirms the results of Hur and Jang [30] that perceived healthiness decreases with anticipated guilt. Hur and Jang [30] found the anticipated guilt associated with a healthy meal (a grilled chicken sandwich on whole-grain bread, mixed salad greens with light Italian dressing, and unsweetened iced tea; with an advertisement including information on health-related attributes) to be 2.835 (*n* = 809), which was closer to our result of the unhealthy product (fried potatoes and sausages): 3.17. In the study of Hur and Jang [30], the mean age of the participants was 33.2 years, and they were mostly (73.4%) white US Americans, compared to 45 years and 100% Finns in our study. These differences in sample materials and the age and cultural background of the participants might explain the differences in expected guilt associated with the sample foods. In addition, interactions between UT belief and other factors might influence the results.

In addition, we found significant interactions between UT belief and GHI, and UT belief and food pleasure orientation when evaluating expected guilt. Expected guilt as regards the unhealthy product was positively related to UT belief only among participants having a lower GHI (Figure 7), and the positive relationship between UT belief and expected guilt of the healthy product was more significant among participants having a lower food pleasure orientation (Figure 8). UT belief seemed to assert an increasingly stronger effect on expected guilt when the other food-related attitudes were weaker.

### 4.5. Interactions between Age and Gender and UT Belief 

In addition to the main effects of UT belief on food expectations, there were quite numerous interactions between UT belief and both the demographic factors and food-related attitudes. The greatest number of interactions were observed between UT belief and age when evaluating the healthy product (see Table S8). The negative effect of UT belief on expected tastiness, healthiness, purchase intention, and positive emotions was more significant among older participants (older than 44 years), while the positive effect of UT belief on expected undesirable nutrients (fat and salt) was more significant among younger participants. In conclusion, age seemed to strengthen the negative effect of UT belief on the expectations of the healthy product.

When evaluating expected nutrient contents, interactions between UT belief and gender were noticed. The positive effect of UT belief on the desirable nutrient (protein) content of the unhealthy product and on the undesirable nutrient (fat) content of the healthy product was especially detected among men, whereas no effect of UT belief on nutrient expectations was identified among women.

### 4.6. Suggestions for Further Studies

We found food pleasure orientation to relate positively to both UT belief and GHI, although UT belief and GHI related negatively to each other. More research is needed to thoroughly explain these complicated relationships between attitudes and beliefs about health, pleasantness, and taste. In addition, the complex interactions of UT belief, GHI, food pleasure orientation, and guilt need to be examined more profoundly.

The reasons for the positive relationship between UT belief and expected guilt need to be investigated more thoroughly. In our research, we observed this relationship with both the unhealthy and the healthy product. Moreover, the possible correlation of UT belief and restrained eating should also be investigated in future studies. Dietary restraint has been associated with guilt both before and after eating and with guilt about food cravings [32,41,42]. Food-related guilt may have positive consequences on eating behavior and lifestyle, such as making healthier food choices and an increase in physical exercise [31]. On the other hand, guilt in combination with dietary restraint may become associated with problematic eating habits, such as regulating guilt through binge eating [41].

Research in authentic real-world contexts is needed to verify the results we found in our online survey. We investigated the expectations associated with two different food products (unhealthy and healthy) and found the effect of UT belief to depend on the health image of the product. More research is needed with different types of food products to explain these complex dependencies and effects related to UT belief. In addition, we noticed numerous interactions between UT belief and both demographic factors and food-related attitudes which need to be investigated more thoroughly. In addition, other factors affecting healthy food choices, such as sustainability and planetary diet, should be investigated in more detail [43,44].

In our study, we explored the strength and effect of explicit UT belief. However, people can hold both an explicit belief and an implicit intuition. Raghunathan et al. [15] have reported that people who implicitly associated unhealthiness and tastiness may explicitly report not agreeing to this belief. However, implicit intuition may be more constant; for example, Mai and Hoffmann [12] noticed the diminishing effect of health consciousness only with explicit UT belief, whereas implicit belief was not changed. More research is needed to explain the relationship between explicit belief and implicit intuition, and the factors affecting these beliefs and intuitions.

## 5. Conclusions

Our findings provide a better understanding of consumers’ attitudes as well as the demographic factors related to UT belief; they also demonstrate the effect of UT belief on food expectations. Consequently, identifying the profiles of UT believers and UT non-believers, will help to develop tailored tools to encourage consumers to make healthier food choices. UT believers and UT non-believers experience health messages differently. Thus, tailoring messages, for example, with a different emphasis on taste and health content for different groups, can produce a better result in terms of healthy food choices cf. [45].

We found a negative relationship between UT belief and health interest, while the relationship of UT belief with male gender and food pleasure orientation was positive. There is a need to diminish the strength of UT belief and its consequent undesirable impact that leads to favoring unhealthy food. This can be achieved through campaigns strengthening health interests and healthy eating; these campaigns should be especially targeted at men. As UT believers are also food-pleasure-oriented consumers, improving the pleasantness of healthy food would tempt them to make healthier food choices.

Our study indicates that a strong UT belief correlates with positive emotions associated with unhealthy food (calmness, satisfaction, energy, and activity) and with negative emotions associated with healthy food (boredom, disgust). Developing healthy food products to evoke more positive emotions instead of negative ones would tempt even more consumers with a strong UT belief to choose healthy foods.

UT belief was found to increase expected food-associated guilt with both the unhealthy and the healthy product. We presume that consumers with higher UT belief are more prone to feeling guilt, since they know they should be eating and liking healthier food. Combining other strong orientations such as a health interest with unhealthy food and a pleasure orientation with healthy food may mitigate the guilt-increasing effect of UT belief.

## Figures and Tables

**Table 1:** The results of the linear regression. The prediction of unhealthy = tasty belief by the demographic factors (gender, age, BMI, education) and attitudes (GHI, food pleasure orientation) (*n* = 1537).

	Unstandardized	Standardized	Sig.	CI (95.0%) for β	
	β	β		Lower Bound	Upper Bound
(Constant)	4.315		<0.001	3.779	4.851
Gender (0 = female; 1 = male)	0.500	0.168 ***	<0.001	0.364	0.637
Age (years)	−0.002	−0.022	0.371	−0.007	0.003
BMI (kg/m^2^)	0.012	0.048 *	0.042	0.000	0.023
Education (0 = no; 1= academic degree)	−0.170	−0.057 *	0.015	−0.307	−0.033
GHI	−0.463	−0.361 ***	<0.001	−0.525	−0.402
Food pleasure orientation	0.111	0.076 **	0.001	0.044	0.177

Model: *R*^2^ = 0.194, adjusted *R*^2^ = 0.190.* *p* < 0.050, ** *p* < 0.010, *** *p* < 0.001.

## Data Availability

The data presented in this study are available on request from the corresponding author. The data are not publicly available due to privacy.

## Supplementary Materials

The following supporting information can be downloaded at: https://www.mdpi.com/article/10.3390/foods11193139/s1, Section S1: The experimental design of the online survey; Section S2: The questionnaire of the online survey; Section S3: The results of linear regression analyses. Prediction of expected attributes of unhealthy product: fried potatoes and sausages (Tables S1–S4); Section S4: The results of linear regression analyses. Prediction of expected attributes of healthy product: vegetable lentil soup (Tables S5–S8).
