# Supplementary material for: “Unhealthy = Tasty”: How Does It Affect Consumers’ (Un)Healthy Food Expectations?
Source: Foods. 2022 Oct 9;11(19):3139. doi: 10.3390/foods11193139 (PMC9563033; doi:10.3390/foods11193139)
Supplement: Supplementary file 1 [file foods-11-03139-s001.zip › foods-1931304-supplementary.pdf]

Section S1

The experimental design of the online survey

Six research groups (R1 – R6) were composed to explore the effect of package labelling (No FoP / FoP) using interventions (No intervention / Intervention 1 / Intervention 2). The number of participants in the different research groups varied from 250 to 262.

**R1**. No intervention + No FoP<br>N = 262	**R3**. Intervention 1 + No FoP<br>N = 253	**R5**. Intervention 2 + No FoP<br>N = 257
**R2**. No intervention + FoP<br>N = 257	**R4**. Intervention 1 + FoP<br>N = 250	**R6**. Intervention 2 + FoP<br>N = 258

FoP: Front of Package label (Heart Symbol) included on the package

Intervention 1: sensory imagery (guided instructions at the beginning of the survey)

Intervention 2: decentering (guided instructions at the beginning of the survey)

The participants:

- To study the strength of Unhealthy = Tasty (UT) belief and the relationship between UT belief and other attitudes (General Health Interest; Food pleasure orientation) and demographic factors (gender, age, body mass index, education), all the research groups (R1 - R6) were involved (N = 1537)
  → Study A

- To study the effect of UT belief on expected food experiences (tastiness, healthiness, purchase intention, nutrient content, emotions), the research group R1 with no intervention and no FoP was involved (N = 262)
  → Study B

Section S2

The on-line survey questionnaire

Part 1. Expectations of the new food products

Look at the product image below for as long as you wish. Then answer the questions using a scale of 1 to 7 (1 = strongly disagree and 7 = strongly agree)

[In the surveys, the order was randomized]

1. I expect this product to taste excellent.
2. I expect that eating this product will be a pleasant experience.

[statements 1 – 2 represented expected tastiness]

3. I expect, that tasting and eating this product…

a. ... calms and reassures me

b. … bores me, does not interest me

c. … satisfies me

d. … makes me associate it with happy memories of childhood

e. … makes me feel guilt

f. … disgusts me

g. … makes me feel active and full of energy

[statements 3a – 3g represented expected emotions associated with the product]

4. In my opinion this product contains a lot of energy.
5. In my opinion this product contains a lot of fat.
6. In my opinion this product contains a lot of salt.
7. In my opinion this product contains a lot of protein.

[statements 4 – 7 represented expected nutrient content]

8. Consuming this product will help me stay fit.
9. In my opinion this product is healthy.
10. This product will help me stay slim.

[statements 8 – 10 represented expected healthiness]

11. I will buy this product (in the future)
12. I prefer this product to other ready-to-eat foods.
13. Next time I buy ready-to-eat foods, I will choose this product.

[statements 11 – 13 represented purchase intention]

Part 2. What kind of food consumer you are?

Using a scale of 1-7 indicate to what extent you agree with following claims.
(scale 1-7: 1 = strongly disagree; 7 = strongly agree)

[In the survey the claims were randomized]

1. Things that are good for me rarely taste good
2. There is no possibility of making food healthier without sacrificing taste
3. Healthy food is usually less tasty

[statements 1 – 3 represented Unhealthy = Tasty belief]

4. Enjoying food is one of the most important pleasure in my life.
5. I would rather eat my favorite meal than watch my favorite television show.
6. I often think about food in a positive anticipatory way
7. Money spent on food is money well spent
8. I have fond memories of family food occasions
9. If I could satisfy my nutritional needs safely, cheaply and without hunger by taking dietary supplements, I would. (Reversed)

[statements 4 – 9 represented Food pleasure orientation]

10. I am very particular about the healthiness of the food I eat.
11. It is important for me that my diet is low in fat.
12. I always follow a healthy and balanced diet.
13. It is important for me that my daily diet contains a lot of vitamins and minerals.
14. The healthiness of food has little impact on my food choices. (Reversed)
15. I eat what I like and I do not worry much about the healthiness of food. (Reversed)
16. The healthiness of snacks makes no difference to me. (Reversed)
17. I do not avoid foods, even if they may raise my cholesterol. (Reversed)

[statements 10 – 17 represented General health interest (GHI)]

Part 3. Background information

Gender

- ☐ Woman
- ☐ Man
- ☐ Other

Year of birth ☐

What is the highest level of education you have completed?

- ☐ No education
- ☐ Comprehensive school
- ☐ High school/vocational education
- ☐ Bachelor degree
- ☐ Master degree
- ☐ Licentiate or doctoral degree

[No education, Comprehensive school, and High school/vocational education represent ‘no academic degree’; Bachelor, Master, and Licentiate or doctoral degree represent an ‘academic degree’]

What is your height (cm)? ☐

What is your weight (kg)? ☐

[Reported height and weight values were used to calculate Body Mass Index (BMI) = kg/m^2 where kg is a person's weight in kilograms and m^2 is their height in meters squared.]

Section S3

The results of the linear regression analyses. The prediction of the expected attributes of the unhealthy product: fried potatoes and sausages (n = 262).

Table S1. The results of the linear regression analyses. The prediction of the expected tastiness, healthiness, and purchase intention of the unhealthy product (fried potatoes and sausages) by the factors: UT belief, GHI, Food pleasure orientation, gender, age, BMI, and education (n = 262).

Expected tastiness_1	Unstandardized β	Standardized β	Sig.	95.0% Confidence Interval for β Lower bound	Upper Bound
(Constant)	1.716		0.039	0.090	3.342
UT belief	0.196	0.167 **	0.007	0.053	0.339
GHI	- 0.191	- 0.123	0.051	- 0.384	0.001
Food pleasure orientation	0.391	0.228 ***	< 0.001	0.199	0.584
Gender (0=female; 1=male)	0.781	0.228 ***	< 0.001	0.392	1.170
Age (years)	0.004	0.039	0.522	- 0.009	0.018
BMI (kg/m^2)	0.021	0.072	0.224	- 0.013	0.055
Education (0=no; 1= academic degree)	- 0.350	- 0.102	0.079	- 0.741	0.041

Model: $R^2 = 0.203$, Adjusted $R^2 = 0.180$. * p < 0.050, ** < p < 0.010, *** p < 0.001.

Expected healthiness_1	Unstandardized β	Standardized β	Sig.	95.0% Confidence Interval for β Lower bound	Upper Bound
(Constant)	0.464		0.482	- 0.835	1.763
UT belief	0.156	0.171 **	0.008	0.042	0.270
GHI	0.061	0.050	0.438	- 0.093	0.214
Food pleasure orientation	0.224	0.168 **	0.005	0.070	0.378
Gender (0=female; 1=male)	0.756	0.284 ***	< 0.001	0.445	1.068
Age (years)	0.004	0.046	0.458	- 0.007	0.015
BMI (kg/m^2)	- 0.013	- 0.056	0.354	- 0.040	0.014
Education (0=no; 1= academic degree)	- 0.177	- 0.066	0.267	- 0.489	0.136

Model: $R^2 = 0.158$, Adjusted $R^2 = 0.134$. * p < 0.050, ** < p < 0.010, *** p < 0.001.

Expected purchase intention_1	Unstandardized β	Standardized β	Sig.	95.0% Confidence Interval for β Lower bound	Upper Bound
(Constant)	1.294		0.098	- 0.240	2.827
UT belief	0.212	0.188 **	0.002	0.077	0.347
GHI	- 0.198	- 0.133 *	0.033	- 0.380	- 0.017
Food pleasure orientation	0.332	0.201 ***	< 0.001	0.151	0.514
Gender (0=female; 1=male)	0.968	0.294 ***	< 0.001	0.601	1.335
Age (years)	- 0.005	- 0.041	0.483	- 0.018	0.008
BMI (kg/m^2)	0.012	0.041	0.479	- 0.021	0.044
Education (0=no; 1= academic degree)	- 0.154	- 0.047	0.411	- 0.524	0.215

Model: $R^2 = 0.232$, Adjusted $R^2 = 0.210$. * p < 0.050, ** < p < 0.010, *** p < 0.001.

Table S2. The results of the linear regression analyses. The prediction of the expected nutrient contents of the unhealthy product (fried potatoes and sausages) by the factors: UT belief, GHI, Food pleasure orientation, gender, age, BMI, and education (n = 262).

**Expected**	**Unstandardized**	**Standardized**			**95.0% Confidence Interval for β**	
**energy content_1**	**β**	**β**		**Sig.**	**Lower bound**	**Upper Bound**
(Constant)	2.430			0.001	0.990	3.870
UT belief	0.009	0.009		0.890	- 0.118	0.136
GHI	- 0.001	- 0.001		0.987	- 0.172	0.169
Food pleasure orientation	0.463	0.316	***	< 0.001	0.293	0.634
Gender (0=female; 1=male)	- 0.204	- 0.070		0.245	- 0.549	0.141
Age (years)	0.010	0.104		0.098	- 0.002	0.022
BMI (kg/m^2)	0.011	0.043		0.480	- 0.019	0.041
Education (0=no; 1= academic degree)	0.245	0.084		0.164	- 0.101	0.592

Model: R^2 = 0.142, Adjusted R^2 = 0.118. * p < 0.050, ** < p < 0.010, *** p < 0.001.

**Expected**	**Unstandardized**	**Standardized**			**95.0% Confidence Interval for β**	
**fat content_1**	**β**	**β**		**Sig.**	**Lower bound**	**Upper Bound**
(Constant)	4.615			< 0.001	3.398	5.833
UT belief	0.017	0.021		0.757	- 0.090	0.124
GHI	0.133	0.124		0.069	- 0.011	0.278
Food pleasure orientation	0.079	0.066		0.284	- 0.066	0.223
Gender (0=female; 1=male)	- 0.387	- 0.163	*	0.010	- 0.678	- 0.095
Age (years)	- 0.001	- 0.008		0.899	- 0.011	0.010
BMI (kg/m^2)	0.011	0.055		0.392	- 0.014	0.037
Education (0=no; 1= academic degree)	0.224	0.094		0.133	- 0.069	0.517

Model: R^2 = 0.070, Adjusted R^2 = 0.044. * p < 0.050, ** < p < 0.010, *** p < 0.001.

**Expected**	**Unstandardized**	**Standardized**			**95.0% Confidence Interval for β**	
**salt content_1**	**β**	**β**		**Sig.**	**Lower bound**	**Upper Bound**
(Constant)	4.211			< 0.001	2.951	5.471
UT belief	0.007	0.009		0.896	- 0.104	0.118
GHI	0.140	0.124		0.065	- 0.009	0.289
Food pleasure orientation	0.089	0.071		0.242	- 0.060	0.238
Gender (0=female; 1=male)	- 0.351	- 0.141	*	0.023	- 0.653	- 0.049
Age (years)	0.001	0.012		0.854	- 0.010	0.012
BMI (kg/m^2)	0.009	0.040		0.521	- 0.018	0.035
Education (0=no; 1= academic degree)	0.456	0.182	**	0.003	0.153	0.759

Model: R^2 = 0.098, Adjusted R^2 = 0.073. * p < 0.050, ** < p < 0.010, *** p < 0.001.

**Expected**	**Unstandardized**	**Standardized**			**95.0% Confidence Interval for β**	
**protein content_1**	**β**	**β**		**Sig.**	**Lower bound**	**Upper Bound**
(Constant)	1.343			0.050	- 0.001	2.688
UT belief	0.166	0.179	**	0.006	0.047	0.284
GHI	0.103	0.084		0.202	- 0.056	0.262
Food pleasure orientation	0.199	0.147	*	0.014	0.040	0.359
Gender (0=female; 1=male)	0.712	0.263	***	< 0.001	0.390	1.034
Age (years)	0.005	0.052		0.408	- 0.007	0.016
BMI (kg/m^2)	0.001	0.003		0.964	- 0.028	0.029
Education (0=no; 1= academic degree)	0.066	0.024		0.690	- 0.258	0.389

Model: R^2 = 0.130, Adjusted R^2 = 0.105. * p < 0.050, ** < p < 0.010, *** p < 0.001.

Table S3. The results of the linear regression analyses. The prediction of the expected emotions associated with the unhealthy product (fried potatoes and sausages) by the factors: UT belief, GHI, Food pleasure orientation, gender, age, BMI, and education (n = 262).

Expected calmness_1	Unstandardized β	Standardized β		Sig.	95.0% Confidence Interval for β Lower bound	Upper Bound
(Constant)	0.858			0.323	- 0.848	2.563
UT belief	0.204	0.171	**	0.008	0.054	0.354
GHI	- 0.078	- 0.049		0.448	- 0.280	0.124
Food pleasure orientation	0.287	0.164	**	0.006	0.085	0.489
Gender (0=female; 1=male)	0.810	0.232	***	< 0.001	0.402	1.218
Age (years)	0.002	0.015		0.812	- 0.013	0.016
BMI (kg/m^2)	0.020	0.067		0.268	- 0.016	0.056
Education (0=no; 1= academic degree)	- 0.316	- 0.090		0.131	- 0.726	0.095

Model: $R^2 = 0.154$, Adjusted $R^2 = 0.131$. * p < 0.050, ** < p < 0.010, *** p < 0.001.

Expected boredom_1	Unstandardized β	Standardized β		Sig.	95.0% Confidence Interval for β Lower bound	Upper Bound
(Constant)	3.959			< 0.001	2.026	5.892
UT belief	0.059	0.046		0.493	- -0.111	0.230
GHI	0.245	0.144	*	0.036	0.017	0.474
Food pleasure orientation	- 0.190	- 0.101		0.104	- 0.419	0.039
Gender (0=female; 1=male)	- 0.265	- 0.071		0.260	- 0.728	0.198
Age (years)	- 0.006	- 0.044		0.504	- 0.022	0.011
BMI (kg/m^2)	- 0.030	- 0.095		0.139	- 0.071	0.010
Education (0=no; 1= academic degree)	0.444	0.118		0.061	- 0.021	0.910

Model: $R^2 = 0.061$, Adjusted $R^2 = 0.035$. * p < 0.050, ** < p < 0.010, *** p < 0.001.

Expected satisfaction_1	Unstandardized β	Standardized β		Sig.	95.0% Confidence Interval for β Lower bound	Upper Bound
(Constant)	1.659			0.058	- 0.055	3.374
UT belief	0.176	0.144	*	0.022	0.025	0.327
GHI	- 0.208	- 0.128	*	0.045	- 0.411	- 0.005
Food pleasure orientation	0.373	0.208	***	< 0.001	0.170	0.576
Gender (0=female; 1=male)	0.895	0.250	***	< 0.001	0.484	1.305
Age (years)	- 0.001	- 0.009		0.883	- 0.016	0.013
BMI (kg/m^2)	0.024	0.078		0.190	- 0.012	0.060
Education (0=no; 1= academic degree)	- 0.220	- 0.061		0.294	- 0.633	0.193

Model: $R^2 = 0.188$, Adjusted $R^2 = 0.165$. * p < 0.050, ** < p < 0.010, *** p < 0.001.

Expected happy memories of childhood_1	Unstandardized β	Standardized β		Sig.	95.0% Confidence Interval for β Lower bound	Upper Bound
(Constant)	- 0.361			0.700	- 2.204	1.482
UT belief	0.256	0.199	**	0.002	0.093	0.418
GHI	0.100	0.059		0.366	- 0.118	0.318
Food pleasure orientation	0.455	0.242	***	< 0.001	0.237	0.674
Gender (0=female; 1=male)	0.725	0.194	**	0.001	0.283	1.166
Age (years)	- 0.002	- 0.017		0.781	- 0.018	0.013
BMI (kg/m^2)	0.021	0.066		0.278	- 0.017	0.060
Education (0=no; 1= academic degree)	0.063	0.017		0.780	- 0.380	0.507

Model: $R^2 = 0.144$, Adjusted $R^2 = 0.121$. * p < 0.050, ** < p < 0.010, *** p < 0.001.

**Expected guilt_1**	**Unstandardized β**	**Standardized β**	**Sig.**	**95.0% Confidence Interval for β Lower bound**	**Upper Bound**
(Constant)	1.031		0.294	- 0.901	2.964
UT belief	0.306	0.231 ***	< 0.001	0.136	0.477
GHI	0.399	0.227 ***	< 0.001	0.170	0.627
Food pleasure orientation	- 0.038	- 0.020	0.741	- 0.267	0.191
Gender (0=female; 1=male)	- 0.517	- 0.133 *	0.029	- 0.980	- 0.054
Age (years)	- 0.025	- 0.190 **	0.003	- 0.041	- 0.009
BMI (kg/m^2)	0.030	0.090	0.146	- 0.010	0.070
Education (0=no; 1= academic degree)	0.380	0.098	0.109	- 0.085	0.845

Model: R^2 = 0.122, Adjusted R^2 = 0.098. * p < 0.050, ** < p < 0.010, *** p < 0.001.

**Expected disgust_1**	**Unstandardized β**	**Standardized β**	**Sig.**	**95.0% Confidence Interval for β Lower bound**	**Upper Bound**
(Constant)	2.329		0.015	0.465	4.193
UT belief	0.066	0.051	0.432	- 0.099	0.230
GHI	0.506	0.296 ***	< 0.001	0.285	0.727
Food pleasure orientation	- 0.173	- 0.092	0.124	- 0.394	0.048
Gender (0=female; 1=male)	- 0.489	- 0.130 *	0.032	- 0.936	- 0.043
Age (years)	- 0.024	- 0.189 **	0.003	- 0.040	- 0.008
BMI (kg/m^2)	0.003	0.008	0.892	- 0.036	0.042
Education (0=no; 1= academic degree)	0.366	0.097	0.110	- 0.083	0.815

Model: R^2 = 0.136, Adjusted R^2 = 0.112. * p < 0.050, ** < p < 0.010, *** p < 0.001.

**Expected energy and activity_1**	**Unstandardized β**	**Standardized β**	**Sig.**	**95.0% Confidence Interval for β Lower bound**	**Upper Bound**
(Constant)	0.876		0.271	- 0.689	2.442
UT belief	0.253	0.228 ***	< 0.001	0.115	0.391
GHI	- 0.102	- 0.069	0.281	- 0.287	0.084
Food pleasure orientation	0.343	0.211 ***	< 0.001	0.157	0.528
Gender (0=female; 1=male)	0.687	0.212 ***	< 0.001	0.312	1.062
Age (years)	0.007	0.065	0.287	- 0.006	0.020
BMI (kg/m^2)	- 0.003	- 0.011	0.852	- 0.036	0.030
Education (0=no; 1= academic degree)	- 0.127	- 0.039	0.507	- 0.504	0.250

Model: R^2 = 0.175, Adjusted R^2 = 0.152. * p < 0.050, ** < p < 0.010, *** p < 0.001.

Table S4. Significant (p < 0.050) interactions with UT belief when evaluating the expected attributes of the unhealthy product (fried potatoes and sausages) (n = 262).

	t	β		Sig.	95.0% Confidence Interval for β Lower bound	Upper Bound
**Expected healthiness_1**						
UT belief * BMI	- 2.230	- 0.021	*	0.027	- 0.040	- 0.003
**Expected protein content_1**						
UT belief * GHI	2.079	0.105	*	0.039	0.006	0.204
UT belief * Gender	3.078	0.337	**	0.002	0.121	0.553
**Expected calmness_1**						
UT belief * Age	- 2.367	-0.012	*	0.019	-0.023	-0.002
**Expected satisfaction_1**						
UT belief * GHI	2.173	0.142	*	0.031	0.013	0.270
**Expected happy memories of childhood_1**						
UT belief * Food pleasure orientation	- 2.040	-0.145	*	0.042	-0.286	-0.005
**Expected guilt_1**						
UT belief * GHI	- 3.051	-0.216	**	0.003	-0.355	-0.077
**Expected energy and activity_1**						
UT belief * Age	- 3.213	-0.015	**	0.001	-0.025	-0.006

Section S4

The results of the linear regression analyses. The prediction of the expected attributes of the healthy product: vegetable lentil soup (n = 262).

Table S5. The results of the linear regression analyses. The prediction of the expected tastiness, healthiness, and purchase intention of the healthy product (vegetable lentil soup) by the factors: UT belief, GHI, Food pleasure orientation, gender, age, BMI, and education (n = 262).

**Expected tastiness_2**	**Unstandardized β**	**Standardized β**	**Sig.**	**95.0% Confidence Interval for β Lower bound**	**Upper Bound**
(Constant)	2.139		0.011	0.488	3.789
UT belief	- 0.144	- 0.126	0.053	- 0.289	0.002
GHI	0.211	0.140 *	0.035	0.015	0.406
Food pleasure orientation	0.278	0.167 **	0.006	0.082	0.473
Gender (0=female; 1=male)	- 0.454	- 0.137 *	0.024	- 0.850	- 0.059
Age (years)	0.005	0.043	0.497	- 0.009	0.019
BMI (kg/m^2)	0.010	0.036	0.560	- 0.024	0.045
Education (0=no; 1= academic degree)	0.116	0.035	0.567	- 0.281	0.513

Model: $R^2 = 0.126$, Adjusted $R^2 = 0.101$. * $p < 0.050$, ** < $p < 0.010$, *** $p < 0.001$.

**Expected healthiness_2**	**Unstandardized β**	**Standardized β**	**Sig.**	**95.0% Confidence Interval for β Lower bound**	**Upper Bound**
(Constant)	2.009		0.005	0.627	3.391
UT belief	0.070	0.075	0.257	- 0.051	0.192
GHI	0.108	0.088	0.193	- 0.055	0.272
Food pleasure orientation	0.324	0.237 ***	< 0.001	0.160	0.487
Gender (0=female; 1=male)	- 0.188	- 0.069	0.264	- 0.519	0.143
Age (years)	0.003	0.031	0.632	- 0.009	0.014
BMI (kg/m^2)	0.008	0.032	0.608	- 0.021	0.037
Education (0=no; 1= academic degree)	0.170	0.062	0.316	- 0.163	0.502

Model: $R^2 = 0.087$, Adjusted $R^2 = 0.062$. * $p < 0.050$, ** < $p < 0.010$, *** $p < 0.001$.

**Expected purchase intention_2**	**Unstandardized β**	**Standardized β**	**Sig.**	**95.0% Confidence Interval for β Lower bound**	**Upper Bound**
(Constant)	0.587		0.493	- 1.097	2.272
UT belief	0.016	0.014	0.827	- 0.132	0.165
GHI	0.420	0.274 ***	< 0.001	0.221	0.619
Food pleasure orientation	0.246	0.145 *	0.016	0.046	0.445
Gender (0=female; 1=male)	- 0.232	- 0.069	0.257	- 0.636	0.171
Age (years)	0.001	0.009	0.890	- 0.013	0.015
BMI (kg/m^2)	- 0.003	- 0.011	0.862	- 0.038	0.032
Education (0=no; 1= academic degree)	0.138	0.041	0.503	- 0.267	0.543

Model: $R^2 = 0.119$, Adjusted $R^2 = 0.095$. * $p < 0.050$, ** < $p < 0.010$, *** $p < 0.001$.

Table S6. The results of the linear regression analyses. The prediction of the expected nutrient contents of the healthy product (vegetable lentil soup) by the factors: UT belief, GHI, Food pleasure orientation, gender, age, BMI, and education (n = 262).

**Expected energy content_2**	**Unstandardized β**	**Standardized β**	**Sig.**	**95.0% Confidence Interval for β Lower bound**	**Upper Bound**
(Constant)	2.171		0.007	0.592	3.750
UT belief	0.026	0.026	0.709	- 0.113	0.165
GHI	0.160	0.118	0.092	- 0.026	0.347
Food pleasure orientation	0.092	0.061	0.336	- 0.095	0.279
Gender (0=female; 1=male)	0.098	0.033	0.611	- 0.280	0.476
Age (years)	- 0.002	- 0.024	0.714	- 0.016	0.011
BMI (kg/m^2)	0.008	0.031	0.635	- 0.025	0.041
Education (0=no; 1= academic degree)	- 0.216	- 0.072	0.264	- 0.596	0.164

Model: R^2 = 0.021, Adjusted R^2 = - 0.006. * p < 0.050, ** < p < 0.010, *** p < 0.001.

**Expected fat content_2**	**Unstandardized β**	**Standardized β**	**Sig.**	**95.0% Confidence Interval for β Lower bound**	**Upper Bound**
(Constant)	1.448		0.022	0.210	2.687
UT belief	0.207	0.248 ***	< 0.001	0.098	0.316
GHI	0.178	0.161 *	0.017	0.032	0.325
Food pleasure orientation	- 0.053	- 0.044	0.474	- 0.200	0.093
Gender (0=female; 1=male)	0.212	0.087	0.161	- 0.085	0.508
Age (years)	- 0.012	- 0.149 *	0.021	- 0.023	- 0.002
BMI (kg/m^2)	0.009	0.042	0.508	- 0.017	0.035
Education (0=no; 1= academic degree)	0.114	0.046	0.453	- 0.184	0.412

Model: R^2 = 0.090, Adjusted R^2 = 0.065. * p < 0.050, ** < p < 0.010, *** p < 0.001.

**Expected salt content_2**	**Unstandardized β**	**Standardized β**	**Sig.**	**95.0% Confidence Interval for β Lower bound**	**Upper Bound**
(Constant)	2.183		0.001	0.881	3.485
UT belief	0.127	0.149 *	0.030	0.013	0.242
GHI	0.170	0.150 *	0.030	0.016	0.324
Food pleasure orientation	- 0.080	- 0.064	0.307	- 0.234	0.074
Gender (0=female; 1=male)	0.103	0.041	0.516	- 0.209	0.415
Age (years)	- 0.008	- 0.098	0.138	- 0.019	0.003
BMI (kg/m^2)	0.008	0.039	0.545	- 0.019	0.036
Education (0=no; 1= academic degree)	0.104	0.042	0.512	- 0.209	0.418

Model: R^2 = 0.041, Adjusted R^2 = 0.014. * p < 0.050, ** < p < 0.010, *** p < 0.001.

**Expected protein content_2**	**Unstandardized β**	**Standardized β**	**Sig.**	**95.0% Confidence Interval for β Lower bound**	**Upper Bound**
(Constant)	1.612		0.036	0.105	3.119
UT belief	0.070	0.071	0.299	- 0.063	0.203
GHI	0.135	0.103	0.136	- 0.043	0.314
Food pleasure orientation	0.057	0.040	0.528	- 0.121	0.236
Gender (0=female; 1=male)	- 0.078	- 0.027	0.671	- 0.439	0.283
Age (years)	0.012	0.119	0.072	- 0.001	0.024
BMI (kg/m^2)	0.015	0.060	0.356	- 0.017	0.046
Education (0=no; 1= academic degree)	- 0.166	- 0.057	0.368	- 0.529	0.196

Model: R^2 = 0.038, Adjusted R^2 = 0.012. * p < 0.050, ** < p < 0.010, *** p < 0.001.

Table S7. The results of the linear regression analyses. The prediction of the expected emotions associated with the healthy product (vegetable lentil soup) by the factors: UT belief, GHI, Food pleasure orientation, gender, age, BMI, and education (n = 262).

**Expected calmness_2**	**Unstandardized β**	**Standardized β**	**Sig.**	**95.0% Confidence Interval for β Lower bound**	**Upper Bound**
(Constant)	1.564		0.060	- 0.067	3.194
UT belief	- 0.078	- 0.070	0.286	- 0.221	0.066
GHI	0.205	0.140 *	0.037	0.012	0.398
Food pleasure orientation	0.340	0.209 ***	< 0.001	0.146	0.533
Gender (0=female; 1=male)	- 0.280	- 0.086	0.159	- 0.671	0.110
Age (years)	0.000	0.000	0.999	- 0.014	0.014
BMI (kg/m^2)	- 0.006	- 0.021	0.736	- 0.040	0.028
Education (0=no; 1= academic degree)	0.220	0.068	0.270	- 0.172	0.613

Model: $R^2 = 0.108$, Adjusted $R^2 = 0.083$. * p < 0.050, ** < p < 0.010, *** p < 0.001.

**Expected boredom_2**	**Unstandardized β**	**Standardized β**	**Sig.**	**95.0% Confidence Interval for β Lower bound**	**Upper Bound**
(Constant)	3.390		< 0.001	1.575	5.205
UT belief	0.241	0.195 **	0.003	0.081	0.401
GHI	- 0.102	- 0.062	0.350	- 0.317	0.113
Food pleasure orientation	0.005	0.002	0.967	- 0.210	0.220
Gender (0=female; 1=male)	0.425	0.117	0.055	- 0.010	0.860
Age (years)	- 0.019	- 0.158 *	0.013	- 0.035	- 0.004
BMI (kg/m^2)	0.012	0.040	0.521	- 0.026	0.050
Education (0=no; 1= academic degree)	- 0.005	- 0.001	0.982	- 0.442	0.432

Model: $R^2 = 0.112$, Adjusted $R^2 = 0.087$. * p < 0.050, ** < p < 0.010, *** p < 0.001.

**Expected satisfaction_2**	**Unstandardized β**	**Standardized β**	**Sig.**	**95.0% Confidence Interval for β Lower bound**	**Upper Bound**
(Constant)	2.031		0.017	0.360	3.703
UT belief	- 0.122	- 0.108	0.104	- 0.269	0.025
GHI	0.190	0.127	0.060	- 0.008	0.388
Food pleasure orientation	0.217	0.131 *	0.032	0.019	0.415
Gender (0=female; 1=male)	- 0.323	- 0.098	0.114	- 0.723	0.078
Age (years)	0.001	0.010	0.873	- 0.013	0.015
BMI (kg/m^2)	0.014	0.048	0.441	- 0.021	0.049
Education (0=no; 1= academic degree)	0.295	0.089	0.149	- 0.107	0.698

Model: $R^2 = 0.095$, Adjusted $R^2 = 0.070$. * p < 0.050, ** < p < 0.010, *** p < 0.001.

**Expected happy memories of childhood_2**	**Unstandardized β**	**Standardized β**	**Sig.**	**95.0% Confidence Interval for β Lower bound**	**Upper Bound**
(Constant)	1.557		0.062	- 0.078	3.191
UT belief	0.068	0.062	0.354	- 0.076	0.212
GHI	0.132	0.091	0.182	- 0.062	0.325
Food pleasure orientation	0.195	0.123 *	0.048	0.002	0.389
Gender (0=female; 1=male)	0.311	0.098	0.119	- 0.081	0.702
Age (years)	- 0.015	-0.140 *	0.033	- 0.029	- 0.001
BMI (kg/m^2)	- 0.021	- 0.077	0.231	- 0.055	0.013
Education (0=no; 1= academic degree)	0.334	0.105	0.096	- 0.060	0.727

Model: $R^2 = 0.064$, Adjusted $R^2 = 0.038$. * p < 0.050, ** < p < 0.010, *** p < 0.001.

Expected	Unstandardized	Standardized		95.0% Confidence Interval for β	
**guilt_2**	**β**	**β**	**Sig.**	**Lower bound**	**Upper Bound**
(Constant)	0.733		0.300	- 0.658	2.125
UT belief	0.288	0.303 ***	< 0.001	0.166	0.411
GHI	0.154	0.122	0.067	- 0.011	0.319
Food pleasure orientation	- 0.039	- 0.028	0.639	- 0.204	0.125
Gender (0=female; 1=male)	0.418	0.150 *	0.014	0.084	0.751
Age (years)	0.000	- 0.002	0.980	- 0.012	0.012
BMI (kg/m^2)	- 0.012	- 0.052	0.402	- 0.042	0.017
Education (0=no; 1= academic degree)	- 0.114	- 0.041	0.502	- 0.449	0.220

Model: R^2 = 0.116, Adjusted R^2 = 0.091. * p < 0.050, ** < p < 0.010, *** p < 0.001.

Expected	Unstandardized	Standardized		95.0% Confidence Interval for β	
**disgust_2**	**β**	**β**	**Sig.**	**Lower bound**	**Upper Bound**
(Constant)	2.858		0.001	1.109	4.608
UT belief	0.258	0.212 **	0.001	0.104	0.412
GHI	- 0.064	- 0.040	0.545	- 0.271	0.143
Food pleasure orientation	- 0.035	- 0.020	0.738	- 0.242	0.172
Gender (0=female; 1=male)	0.545	0.154 *	0.011	0.127	0.964
Age (years)	- 0.023	- 0.193 **	0.002	- 0.038	- 0.008
BMI (kg/m^2)	0.008	0.026	0.675	- 0.029	0.044
Education (0=no; 1= academic degree)	- 0.007	- 0.002	0.974	- 0.428	0.414

Model: R^2 = 0.141, Adjusted R^2 = 0.117. * p < 0.050, ** < p < 0.010, *** p < 0.001.

Expected	Unstandardized	Standardized		95.0% Confidence Interval for β	
**energy and activity_2**	**β**	**β**	**Sig.**	**Lower bound**	**Upper Bound**
(Constant)	1.791		0.031	0.161	3.420
UT belief	- 0.042	- 0.038	0.567	- 0.185	0.102
GHI	0.252	0.174 *	0.011	0.059	0.444
Food pleasure orientation	0.189	0.118	0.055	- 0.004	0.382
Gender (0=female; 1=male)	- 0.346	- 0.109	0.082	- 0.736	0.044
Age (years)	- 0.005	- 0.051	0.434	- 0.019	0.008
BMI (kg/m^2)	0.020	0.075	0.239	- 0.014	0.055
Education (0=no; 1= academic degree)	0.089	0.028	0.654	- 0.303	0.482

Model: R^2 = 0.075, Adjusted R^2 = 0.049. * p < 0.050, ** < p < 0.010, *** p < 0.001.

Table S8. Significant ($p < 0.050$) interactions with UT belief when evaluating the expected attributes of the healthy product (vegetable lentil soup) ($n = 262$).

	t	β	Sig.	95.0% Confidence Interval for β Lower bound	Upper Bound
**Expected tastiness_2**					
UT belief * Age	- 2.395	- 0.012 *	0.017	- 0.022	- 0.002
UT belief * Education	- 2.004	- 0.276 *	0.046	- 0.548	- 0.005
**Expected healthiness_2**					
UT belief * Age	- 2.588	- 0.011 *	0.010	- 0.019	- 0.003
**Expected purchase intention_2**					
UT belief * Age	- 2.607	- 0.013 *	0.010	- 0.023	- 0.003
**Expected fat content_2**					
UT belief * Age	- 2.961	- 0.011 **	0.003	- 0.018	- 0.004
UT belief * Gender	2.677	0.271 **	0.008	0.072	0.470
UT belief * BMI	- 2.077	- 0.018 *	0.039	- 0.035	- 0.001
**Expected salt content_2**					
UT belief * Age	- 2.462	- 0.009 *	0.014	- 0.017	- 0.002
UT belief * BMI	- 2.104	- 0.019 *	0.036	- 0.037	- 0.001
**Expected protein content_2**					
UT belief * Age	- 3.306	- 0.014 **	0.001	- 0.023	- 0.006
UT belief * Food pleasure orientation	- 2.739	- 0.158 **	0.007	- 0.271	- 0.044
**Expected calmness_2**					
UT belief * GHI	1.992	0.120 *	0.047	0.001	0.238
**Expected satisfaction_2**					
UT belief * Age	- 2.911	- 0.014 **	0.004	- 0.024	- 0.005
**Expected happy memories of childhood_2**					
UT belief * Age	- 2.839	- 0.014 **	0.005	- 0.023	- 0.004
**Expected guilt_2**					
UT belief * Food pleasure orientation	- 2.900	- 0.155 **	0.004	- 0.260	- 0.050
**Expected energy and activity_2**					
UT belief * Age	- 2.229	- 0.011 *	0.027	- 0.020	- 0.001
